# The adenovirus oncoprotein E1B-55K reshapes epigenetic histone modifications in primary human cells

**DOI:** 10.1128/mbio.03470-25

**Published:** 2026-01-26

**Authors:** Konstantin von Stromberg, Laura Seddar, Britta Gornott, Thomas Dobner, Luca D. Bertzbach, Wing-Hang Ip

**Affiliations:** 1 Leibniz Institute of Virology (LIV) 28367, Hamburg, Germany; Princeton University, Princeton, New Jersey, USA

**Keywords:** adenovirus, chromatin, epigenetics, H3K4me3, H3K27ac, histone modifications, oncogene, transduction

## Abstract

Oncogenic viruses can induce epigenetic changes in host cells, which may contribute to cell transformation and tumorigenesis by altering gene expression patterns. Human adenoviruses, while primarily known for their lytic infections, have also been implicated in oncogenic transformation in experimental settings, but the mechanisms underlying adenovirus oncoprotein-induced epigenetic dysregulation remain poorly defined. Building on our recent findings that the adenoviral oncoprotein E1B-55K coordinates cellular transformation through interactions with DNA-bound host transcription factors, we next sought to determine whether these interactions also influence the chromatin landscape. For this purpose, we investigated the epigenetic consequences of E1B-55K expression in primary human mesenchymal stromal cells using histone-targeting MNase chromatin immunoprecipitation-seq, complemented by RNA-seq analysis. We demonstrate that stable expression of HAdV-C5 E1B-55K leads to widespread changes in histone post-translational modifications across the genome. Most notably, E1B-55K expression results in a marked loss of the activating histone marks H3K4me3 and H3K27ac at specific promoters and enhancers. These epigenetic alterations correspond with significant changes in gene expression, suggesting that E1B-55K interferes with normal transcriptional programming by altering the establishment of key histone modifications. Our findings help explain how E1B-55K drives viral transformation by changing epigenetic patterns and improving our understanding of virus-host interactions at the chromatin level. By describing how human adenoviruses disrupt epigenetic homeostasis, we provide insights into the mechanisms by which oncogenic viruses promote early events in cellular transformation.

**IMPORTANCE:**

Oncogenic viruses can reshape host cell epigenomes to promote transformation, yet the mechanisms by which adenoviral oncogenes exert such control remain poorly understood. Here, we reveal that the human adenovirus E1B-55K oncoprotein induces widespread loss of activating histone modifications, leading to transcriptional silencing of key cellular genes in primary human cells. Building on our previous demonstration of efficient adenoviral transformation of primary human mesenchymal stromal cells, this study provides the first comprehensive view of E1B-55K-mediated epigenetic reprogramming. Our findings uncover a link between E1B-55K-host transcription factor interactions and chromatin remodeling, offering new insight into how adenoviruses disrupt epigenetic homeostasis to initiate early events in oncogenic transformation. These results provide direct evidence for the transcriptional repression function long postulated for E1B-55K and advance understanding of virus-host interplay at the chromatin level.

## INTRODUCTION

Tumor viruses are believed to account for at least ~10% of the global cancer burden (1, 2). Among these are the DNA tumor viruses, including the human papillomavirus (HPV), Merkel cell polyomavirus, Kaposi’s sarcoma-associated herpesvirus, hepatitis B virus, adenoviruses, and Epstein-Barr virus (3). The associated tumors, such as cervical cancer in the case of HPV, can contain integrated viral genomes or high baseline levels of viral oncogenes like E6 and E7 (4). These oncogenes can interfere with the host cell machinery in various ways, including epigenetic regulation of host gene expression.

Epigenetics is the study of heritable changes that are induced by alterations of the chromatin structure, which affects regulatory access to the DNA (5). One avenue of such control is the post-translational modification (PTM) of histone tails, such as tri-methylation of histone 3 at lysine residue 4 (H3K4me3) or the acetylation of histone 3 at lysine residue 27 (H3K27ac), both of which are strongly associated with the transcriptional activation of associated genes. Likewise, repression of chromatin access can be controlled via the facultative heterochromatin modification H3K9me3 or the constitutive heterochromatin modification H3K27me3 (6–8). Other avenues include DNA methylation, usually associated with silenced genes, or various non-coding RNA transcripts. Many studies have shown aberrant DNA methylation patterns in cervical cancers or dysregulation of histone PTMs as a consequence of interactions with specific transcription factors (9–12).

Adenoviruses have served as important model systems for the discovery and study of integral biological pathways, such as the p53 network (13, 14). Their oncoproteins, particularly E1A and E1B-55K, have been instrumental in uncovering fundamental mechanisms of viral oncogenesis and cellular regulation (15–17). The primary mitogenic driver of adenovirus is the *E1A* oncogene, whose expression reprograms the host genome by displacing Rb family proteins and recruiting p300/CBP acetylases to activate S phase genes, while globally redistributing histone acetylation (18–20). The anti-apoptotic E1B-55K, however, has been largely overlooked regarding its effect on the host mRNA expression machinery. We and others recently demonstrated that E1B-55K represses transcription by binding to p53 and other transcription factors on the host chromatin to deregulate associated genes to favor virus replication and inhibit apoptosis; nevertheless, the underlying mechanisms remain elusive (21–23). For p53, E1B-55K employs diverse strategies—direct binding to block transcription, promoting proteasomal degradation via an E4orf6-containing E3 ubiquitin ligase, or relocalizing the factor itself (16, 17). In addition, accumulating evidence suggests that E1B-55K affects transcription via epigenetic mechanisms, an idea supported by studies in the context of the viral genome, where E1B-55K counteracts cellular chromatin remodelers such as SPOC1, Daxx/ATRX, and KAP1 (24–26). To further investigate how these protein-DNA interactions affect epigenetic processes, we used human mesenchymal stromal cells (hMSCs), which can be efficiently transformed by the continuous expression of adenoviral E1A and E1B-55K oncogenes (27). Using this system, we examined whether E1B-55K plays a role in modulating the host epigenetic landscape. We generated hMSCs expressing different combinations of adenovirus oncogenes to assess their effects in an early transformation context. Our analysis of the epigenetic landscape in hMSCs uncovered that E1B-55K inhibits activating histone PTMs to dysregulate transcription at specific genomic loci. These findings provide critical insights into one of the mechanisms by which E1B-55K disrupts host transcriptional control, thereby advancing our understanding of adenoviral oncogenesis and the dynamic interplay between viral proteins and the host epigenetic landscape (28).

## RESULTS

### Stable expression of adenoviral oncogenes in hMSCs via transduction and FACS-based selection

**Fig 1:**
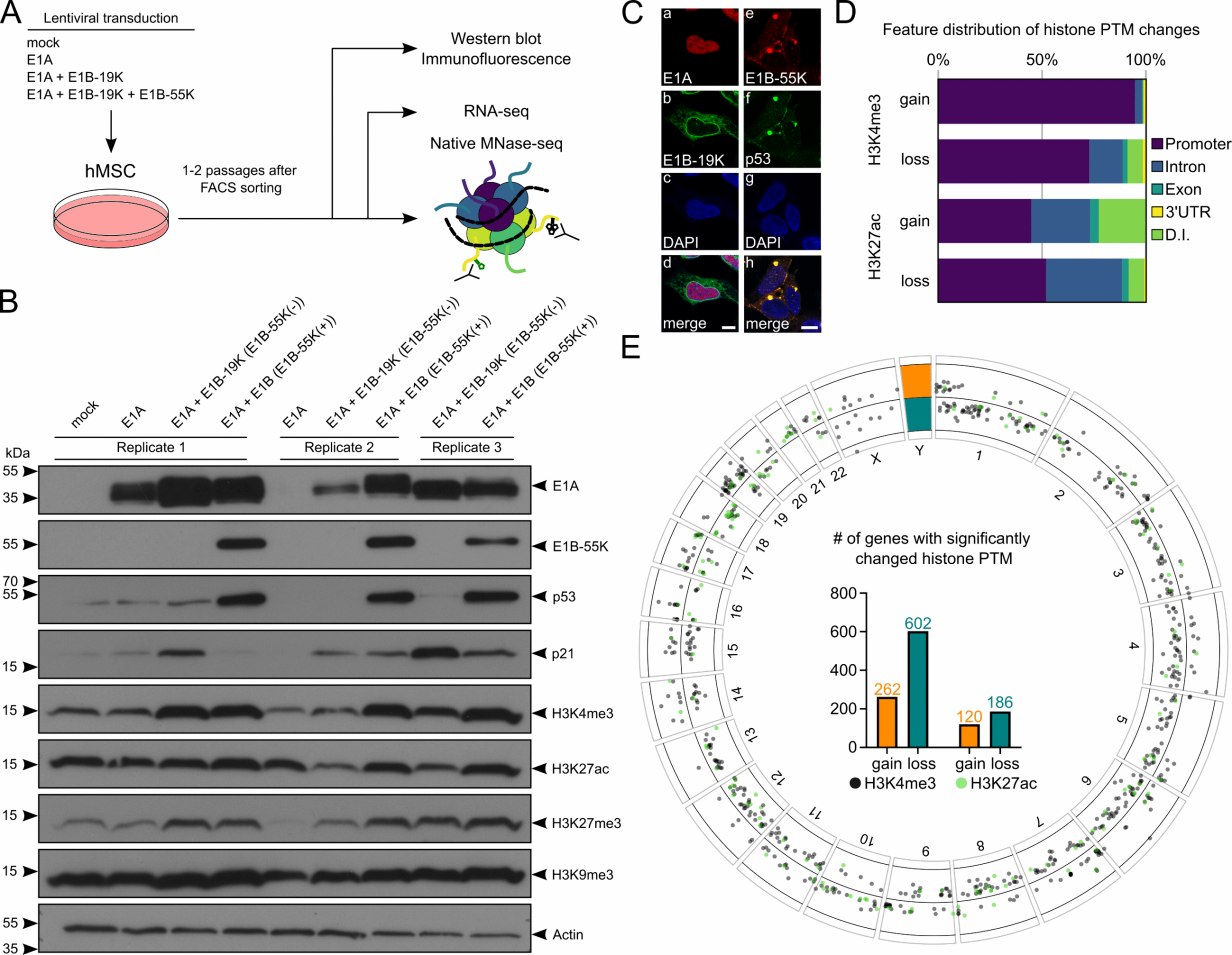
Tracing the effect of adenoviral oncogenes on primary hMSCs. (**A**) Schematic overview of the RNA-seq analysis and epigenetic profiling multiomics workflow, combined with western blotting and immunofluorescence in early-passage (1–2 post-sorting) hMSCs, comparing conditions with and without adenoviral E1B-55K wild-type expression following lentiviral transduction. (**B**) Western blot analysis showing steady-state protein levels in transduced primary hMSCs. (**C**) Immunofluorescence images of transduced primary hMSCs showing expression of adenoviral proteins E1A, E1B-19K, E1B-55K, and cellular p53 as indicated. DAPI was used to visualize the nucleus. Scale bars: 10 µm. (**D**) Feature distribution of PTM sites from panel E, categorized by genomic region and direction of modification. UTR, untranslated region; D.I., distal intergenic. (**E**) Genomic distribution of differentially modified histone PTM sites, along with the total number of associated genes exhibiting gained or lost H3K4me3 (black) or H3K27ac (green) marks. As no peaks were found on the Y-chromosome, we chose to use it to visualize the color code with orange indicating gained and petrol indicating lost histone PTM.

With this study, we build on our earlier work (22, 27) by transducing primary hMSCs from three donors (replicates 1–3) using HAdV-C5 early oncogenes E1A and E1B. Our aim was to investigate the potential mechanisms underlying E1B-55K-mediated gene repression. It has long been known that the adenoviral E1A protein can actively remodel host chromatin, thereby interfering with antiviral gene expression (19, 20) and can directly inhibit the p300 histone acetyltransferase (18). The influence of E1B-55K on the epigenetic landscape, on the other hand, was first proposed over 30 years ago by Yew et al. (21) and Martin and Berk (29). It binds to p53 and suppresses its ability to mediate cell cycle arrest and apoptosis (16, 17, 22, 30). Given these findings, we specifically sought to identify epigenetic alterations at target gene loci indicative of such regulatory activity. Primary hMSCs were efficiently transduced and expanded under E1A + E1B-19K (E1B-55K [−]) or full-length E1A + E1B (E1B-55K [+]) conditions, while control cells (mock or E1A-only) failed to propagate (Fig. 1A) (31). Western blotting confirmed robust viral oncoprotein expression and consistent E1B-55K-dependent repression of the p53 pathway (Fig. 1B), as p21 levels were reduced in the presence of E1B-55K. We observed that global levels of both H3K4me3 and H3K27ac were slightly increased in the E1B-55K (+) condition in replicates 2 and 3, while the other histone PTMs were largely comparable (Fig. 1B). Immunofluorescence further validated expected localization patterns of E1A, E1B-19K, E1B-55K, and p53 (32–36), demonstrating successful establishment of the model system (Fig. 1C).

### E1B-55K-dependent modulation of H3K4me3 and H3K27ac histone modifications in hMSCs

**Fig 2:**
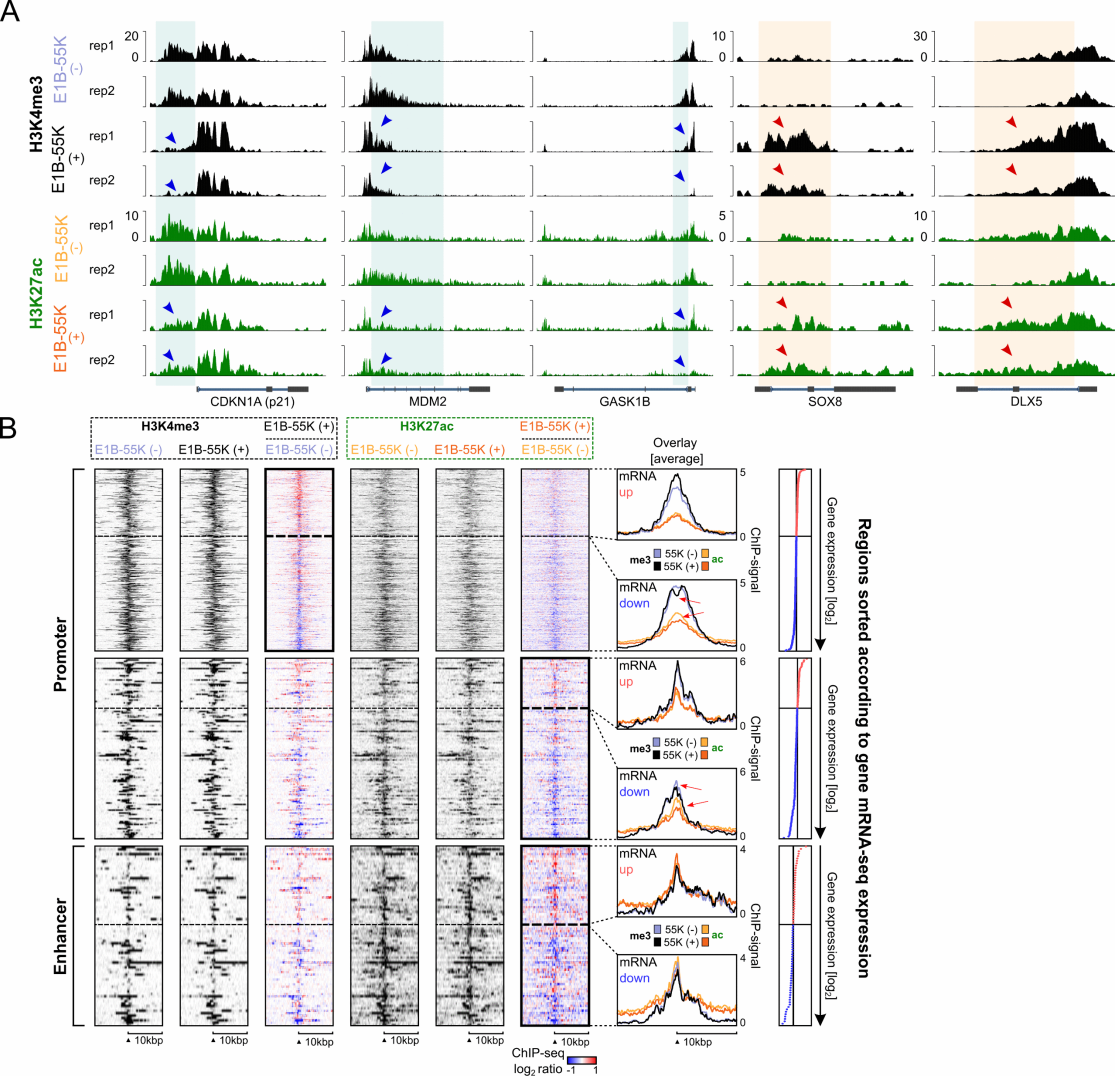
Comparing gene expression and histone chromatin immunoprecipitation (ChIP)-seq signal of associated differential histone PTM regions. (**A**) Visualization of five significantly altered regions identified by differential analysis. Three regions (highlighted with green boxes and blue arrowheads) show loss of both H3K4me3 and H3K27ac signals in the presence of E1B-55K and are associated with p53 target genes. The remaining two regions (highlighted with orange boxes and red arrowheads) exhibit increased signal in the presence of E1B-55K. The y-axis represents the ChIP-seq signal intensity for each histone mark. (**B**) MNase ChIP-seq heatmaps displaying histone PTMs at regulatory regions in E1B-55K (+) versus E1B-55K (−) hMSCs with associated genes and their corresponding mRNA log₂ fold changes, which were used to sort from top (upregulated, red dots) to bottom (downregulated, blue dots). The heatmaps visualize the indicated (bold border) gene set with plots representing promoter-associated H3K4me3 (top row) or H3K27ac (middle row) as well as enhancer-associated H3K27ac (bottom row). The latter region set was defined by overlapping differential non-promoter peaks with the FANTOM5 human enhancer data set (37–39). The ratiometric plots show the log₂ difference in ChIP-seq signal between E1B-55K (+) and (−) conditions, with red representing gained signal and blue representing lost signal. Overlay plots show the average ChIP-seq signal tracks with red arrows highlighting a representative loss of histone modification in E1B-55K (+) hMSCs.

Next, we combined MNase- and RNA-seq data of primary hMSC early post-transduction to identify the influence of E1B-55K on chromatin accessibility patterns and transcriptional activity (Fig. S1). The majority of differential H3K4me3 peaks were located in promoter regions, while H3K27ac peaks were also observed in enhancers and other intergenic regions (Fig. 1D). Both H3K4me3 and H3K27ac are typically enriched in promoter regions of actively transcribed genes, appearing as sharp, narrow peaks near transcription start sites. We annotated differential regions based on the nearest gene and illustrated their chromosomal distribution in Fig. 1E. We found more genes that lost a significant amount of H3K4me3 and H3K27ac in their regulatory regions than genes that have gained these PTMs. Interestingly, some chromosomal regions were clustering large amounts of either gained (chromosome 19 and 21) or lost signal (chromosome 5 and 13). These results indicate a general repressive effect of E1B-55K on chromatin activity, consistent with its previously described role in transcriptional repression (21, 22). Representative gene tracks are shown in Fig. 2A, highlighting a marked reduction in the activating histone modifications H3K4me3 and H3K27ac at three canonical p53 target genes: *CDKN1A* (*p21*), *MDM2*, and *GASK1B*. Likewise, some genes have gained these modifications, such as *SOX8* and *DLX5* when E1B-55K is present in the cells.

The heatmaps in Fig. 2 display a 20 kb window around all significantly altered promoter-associated H3K4me3 (Fig. 2B, top) or H3K27ac (Fig. 2B, middle), as well as enhancer-associated H3K27ac regions (Fig. 2B, bottom). These regions were sorted according to the expression levels of their associated genes. Ratiometric heatmaps illustrate the signal differences in histone PTMs between E1B-55K (−) and (+) conditions. The differences largely correspond to changes in gene expression, which were used to sort the heatmap from top (upregulated) to bottom (downregulated), and were particularly prominent in promoter-associated H3K4me3 regions. On the far right of each panel, average signal plots were overlaid and grouped by up- or downregulated genes. Given that the biological interplay between H3K4me3 and H3K27ac is not well described yet, we aimed to assess whether observed changes in one modification were mirrored in the other, or whether distinct changes were specific to each mark (40–42). This may help deepen our understanding of the mechanism by which E1B-55K affects histone PTMs. Regions with increased H3K4me3 signal in cells expressing E1B-55K associated with upregulated genes showed a clear gain in H3K4me3 but minimal change in H3K27ac (Fig. 2B, upper right, up). In contrast, regions linked to downregulated genes displayed a marked reduction in both H3K4me3 and H3K27ac signals at their centers (Fig. 2B, upper right, down; red arrows). We observed a similar pattern in promoter-associated H3K27ac regions (Fig. 2B, middle right), with both up- and downregulated genes showing correlated changes in histone PTMs (red arrows). However, the trend was less distinct in enhancer-associated H3K27ac regions, likely due to the inherent difficulty of definitively linking enhancers to their target genes (43). While we could not directly demonstrate that E1B-55K is the sole cause of each individual histone modification change, the observed patterns were consistent with a more repressive chromatin phenotype, as suggested by earlier studies (44, 45).

### E1B-55K-mediated repression of tumor-associated pathways in hMSCs

**Fig 3:**
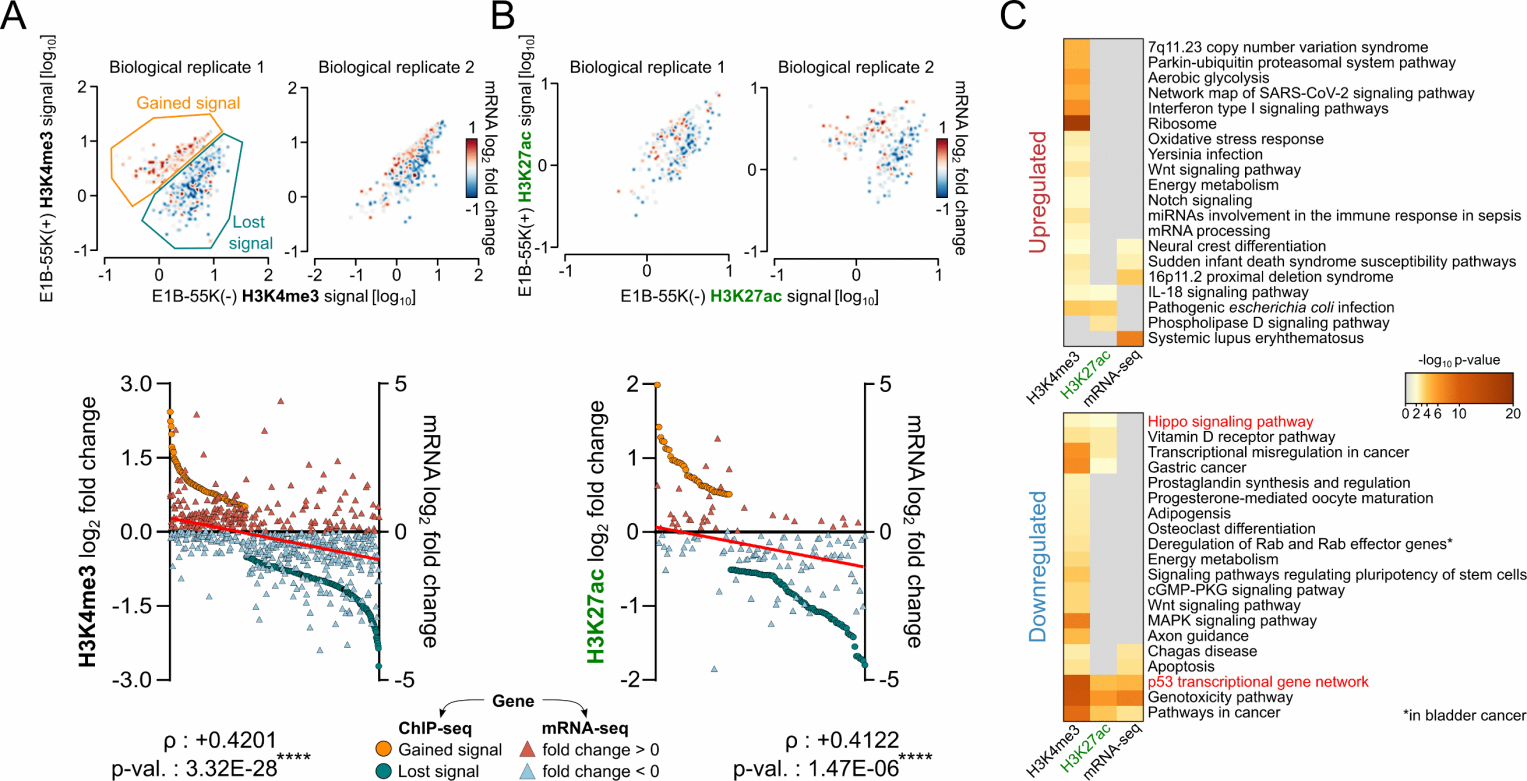
Assessing the influence of E1B-55K expression on epigenetic and transcriptomic repression of tumorigenic pathways. Differential analysis of H3K4me3 (**A**) and H3K27ac (**B**) ChIP-seq signals gained (orange) or lost (petrol) in E1B-55K (+) hMSCs compared to E1B-55K (−) cells. The upper plots show the signal in the respective replicates (hMSCs from different donors). Here, the ChIP-seq signal [log_10_] was quantified within a 1 kb window around significantly altered regions. The corresponding log₂ fold change in mRNA expression of the nearest gene is shown using a color scale from blue (downregulated) to red (upregulated). The lower plots show the correlation between changes in promoter-associated histone PTMs and associated gene expression. Pearson correlation coefficient (ρ) indicates the linear relationship between histone PTM changes, as identified and quantified by diffReps, of H3K4me3 and H3K27ac [log_2_] and mRNA expression levels [log_2_] (red triangles: upregulated; blue triangles: downregulated). Statistical significance was determined using a two-tailed *t*-test; ****P < 0.0001. (**C**) Metascape pathway enrichment analysis of combined upregulated (top) and downregulated (bottom) gene sets, grouped by corresponding histone PTM and gene expression changes. Enriched terms related to the Hippo signaling pathway and p53 transcriptional network are highlighted in red.

To assess the functional consequences of E1B-55K-induced changes in activating histone PTMs, we associated regions with gains or losses in histone PTMs to the corresponding mRNA log_2_ fold change of their nearest genes (Fig. 3A and B). We performed Pearson correlation coefficient (ρ) analyses on the relationship between the differential chromatin immunoprecipitation (ChIP)-seq signal log_2_ fold value and the nearest gene mRNA log_2_ fold value (Fig. 3A and B), which correlated moderately positive with a ρ of 0.4201 (*P*-value 3.32 × 10^−28^) and 0.4122 (*P*-value 1.47 × 10^−6^), respectively. Owing to this general correlation, we combined the histone PTM gene sets, as annotated in Fig. 3C, with all significantly E1B-55K-mediated up- or downregulated genes (adjusted *P*-value ≤ 0.01) and performed Metascape-based pathway analysis. Interestingly, several of the genes showing E1B-55K-dependent upregulation of H3K4me3 were categorized into diverse immune- and infection-related pathways (Fig. 3C, top), such as “interferon type I signaling pathways,” “IL-18 signaling pathway,” and “miRNA involvement in the immune response in sepsis”—while several others were associated with metabolic functions. However, no clear connection with upregulated mRNA could be identified. Pathways related to cellular metabolism and ribosomal biogenesis were also found to be enriched with H3K4me3 modification, a potential side effect of a non-functional p53 pathway, allowing cells to retain a mitogenic state, as explained by the increased need for enhanced stimulation of protein synthesis (46). The most prominent cluster of downregulated transcripts that positively correlates with reduced levels of the histone modifications H3K4me3 and H3K27ac (Fig. 3C, bottom) included genes involved in the interconnected “p53 transcriptional gene network,” “pathways in cancer,” and “genotoxicity” pathways. These pathways have been linked to E1B-55K function by our group and others (22, 47). Additionally, we observed decreased H3K4me3 and H3K27ac signals at promoter regions of genes within the Hippo signaling pathway, which is regulated by the TEAD family of transcription factors (48). However, this epigenetic change did not translate into a significant repression of gene expression at the transcriptomic level. We were also interested in whether E1B-55K can influence facultative or constitutive heterochromatin, represented by H3K27me3 and H3K9me3, respectively. While we could identify some significant changes in these repressing marks, we could not correlate this to changes in mRNA expression of the nearest genes (Fig. S2). Nevertheless, we conducted biological pathway analysis of the associated genes and found no significant enrichment for H3K9me3-marked regions. In contrast, H3K27me3-associated genes revealed significant enrichment in multiple pathways, many of which were related to neurological differentiation processes (Fig. S2).

## DISCUSSION AND CONCLUSIONS

In this study, we observed a widespread loss of ChIP-seq signal in numerous genomic regions upon E1B-55K expression, supporting the hypothesis that E1B-55K acts as a genome-wide transcriptional repressor. Remarkably, some of these regions exhibit clustering based on their signal change (Fig. 1E). For example, on chromosome 21, all identified regions showed a gain in signal, whereas on chromosome 13, nearly all regions displayed a loss in signal. This pattern suggests that E1B-55K may exert long-range regulatory effects on genes organized within topologically associating domains (TADs), pointing to a potential direction for future research (49). TAD boundaries are enriched in CTCF binding sites (50)—a protein previously found to be specifically enriched by the HAdV-A12 E1B-55K protein (22) and is known to play an important role in adenoviral DNA replication (51).

As expected, the most prominent regions exhibiting a loss of activating histone PTMs are associated with p53-regulated pathways, as shown in Fig. 2A and 3C. Notably, the promoter regions of MDM2 and CDKN1A displayed the most significant loss of histone PTM signal. Transcriptional activation of p53 target genes is known to involve recruitment of coactivators such as the histone acetyltransferase p300 or the MLL3-containing methyltransferase complex (52, 53). Since E1B-55K has been shown to interact strongly with DNA-bound transcription factors (22), it is plausible that it disrupts recruitment of these coactivator complexes, thereby reducing activating histone marks (see Fig. 2A). Additionally, the observed repression could mirror the action of the BOZF-1 repressor, which binds to Sp1-rich GC boxes upstream of the CDKN1A transcription start site and blocks access to the p53-responsive enhancer (54). Consistent with this, the most significantly affected biological pathways center on p53, cancer, and genotoxic stress responses (Fig. 3C). Among the upregulated pathways, the “ribosome” pathway was most significantly enriched, marked by overexpression of several ribosomal protein-encoding genes. This may reflect a failure to repress the p53 response in cells expressing E1A but lacking E1B-55K, which can trigger senescence and growth arrest (55). In such cells, ribosomal gene expression is known to be downregulated (56, 57). We also observed increased H3K4me3 signals in several immune-related pathways, though with lower statistical significance. This effect may likewise stem from a dysregulated p53 response, as p53 has established roles in modulating immune-related genes (58). Interestingly, genes within the Hippo signaling pathway showed loss of both activating histone marks (H3K4me3 and H3K27ac) without corresponding changes in mRNA expression. This loss in histone PTMs might result from interference with TEAD and AP-1 transcription factors, as previously described (22, 59). The reduction supports a possible synergistic repression mediated by both E1A and E1B-55K (60). It is possible that E1B-55K reduces H3K27ac and H3K4me3 levels by recruiting chromatin-modifying enzymes to DNA, such as histone deacetylases or demethylases. Consistent with this idea, E1B-55K has been shown to interact with components of HDAC complexes, including HDAC1 and Sin3A, and proteomic studies have identified multiple chromatin-associated factors among E1B-55K interactors (16, 61).

Regarding heterochromatin-associated histone PTMs, we identified several genomic regions that showed changes in signal intensity. However, a clear link between these changes and the expression of nearby genes could not be reliably established (Fig. 2; Fig. S2). This may be due to the nature of the H3K9me3 mark: its width and distribution can vary significantly depending on the chromatin context and the cell’s developmental stage (62). In contrast, marks like H3K4me3 and H3K27ac are more consistently localized to narrow promoter and enhancer regions, making it easier to associate them with specific gene expression changes. This variability complicates the interpretation of H3K9me3 changes, as we were unable to identify any significantly enriched pathways among genes exclusively gaining or losing this mark. Likewise, differential changes in H3K27me3 in hMSCs expressing E1B-55K only revealed pathway enrichment related to neuronal differentiation (Fig. S2B), aligning with previous studies that link H3K27me3 to neuronal development (63, 64).

It is known that cells are more efficiently transformed using the full E1 region (65). Since we compared cells that express the full E1 region with cells that lack E1B-55K, but still express E1A, it is conceivable that our analysis inadvertently compared two different stages of early transformation. Thus, the co-expression of both E1B proteins could accelerate or intensify the transformation process, potentially accounting for the observed epigenetic changes without implying a direct role for E1B-55K in regulating heterochromatin-associated PTMs. Our findings may therefore reflect a more advanced stage of cellular transformation rather than direct interference with heterochromatin (37, 38). We conclude that E1B-55K primarily affects activating epigenetic marks such as H3K4me3 and H3K27ac, likely through interactions with DNA-bound transcription factors, in addition to its established role in p53 sequestration. Future studies are needed to further clarify the link between ChIP-seq and mRNA-seq findings and to understand the downstream consequences of these epigenetic modifications. Moreover, since numerous histone PTMs remain unexamined in this study, it is possible that E1B-55K has a more complex impact yet to be studied.

In summary, our data support a previously hypothesized function of large adenoviral E1B proteins: interaction with DNA-bound transcription factors to modulate the host epigenetic landscape. This activity contributes to the suppression of antiviral responses and facilitates cellular transformation. However, the detailed mechanism of transcriptional repression by E1B-55K remains an open question for future investigation.

Building upon our recent work on adenovirus-induced cell transformation (22, 27), this study further expands the spectrum of regulatory functions attributed to the adenoviral E1B-55K protein. While E1B-55K is classically recognized for its ability to repress p53, prior findings, including its interactions with cellular regulators such as KAP1 and its involvement in SUMO-dependent control of host processes, already point to a broader impact on cellular regulation (26, 66). Here, we extend these insights by demonstrating that E1B-55K also influences the chromatin landscape, thereby integrating multiple modes of host gene regulation into its transforming activity (21, 25).

## MATERIALS AND METHODS

### Cells

Bone marrow-derived hMSCs (27, 67) were cultured in Alpha Minimum Essential Medium (α-MEM; Merck) containing 1 g/L D-glucose and 2.2 g/L sodium bicarbonate. The medium was supplemented with 5% (vol/vol) human platelet lysate supernatant (provided by Claudia Lange [67]), 0.04% heparin (heparin-sodium, 200,000 I.U., Braun) and 2 mM L-alanyl-L-glutamine dipeptide (GlutaMAX Supplement, 100×, Thermo Fisher Scientific). Cells were maintained at 37°C in a humidified atmosphere with 5% CO_2_. Routine testing for mycoplasma contamination was performed using the PCR Mycoplasma Test Kit I/C (PromoKine).

### Lentiviral vectors

Lentiviral gene ontology vectors (68) were used to deliver adenoviral oncogene sequences. The lentiviral vectors and associated primers have been described previously (22). The sequences of full-length HAdV-C5 E1A (GenBank accession no. AY339865, nucleotides [nt] 560–1,545) and full-length E1B (GenBank accession no. AY339865, nt 1,714–3,630), including the first nucleotides of the pIX gene, were used in this study. The E1B-55K (−) construct contains four stop codons within the E1B coding region (32).

### hMSC transductions

Cells for transduction were seeded in six-well plates using the appropriate fully supplemented culture medium and grown to 70%–80% confluency. Prior to transduction, the medium was aspirated and replaced with 1 mL of fresh, fully supplemented medium containing 20 mM HEPES (4-[2-hydroxyethyl]−1-piperazineethanesulfonic acid; Sigma-Aldrich) and 8 μg/mL Polybrene (Merck Millipore) per well. Lentiviral supernatant (500 μL) was added dropwise to each well, and plates were gently swirled to ensure even distribution. The plates were then centrifuged at 2,000 rpm at room temperature for 45 min, followed by incubation at 37°C for 24 h. This transduction step was repeated once after 24 h. For subsequent fluorescence-activated cell sorting (FACS), the culture medium was removed, and cells were washed twice with sterile phosphate-buffered saline (PBS). Cells were then harvested as previously described, resuspended in 500 μL of PBS containing 1% FBS, and passed through a sterile 5 mL Falcon round-bottom polystyrene tube with a cell strainer cap (Stemcell Technologies). Cell sorting was performed at the in-house FACS facility using a BD FACSAria Fusion flow cytometer (BD Biosciences), based on Venus and BFP fluorescence expression. Post-sorting, cells were pelleted, washed, and seeded into the appropriate culture plates. The culture medium was refreshed daily for the following weeks.

### Immunoblotting

Cells were lysed in radioimmunoprecipitation assay buffer (50 mM Tris-HCl, pH 8.0; 150 mM NaCl; 5 mM EDTA; 1 mM dithiothreitol; 0.1% sodium dodecyl sulfate; 1% Nonidet P-40; 0.1% Triton X-100; and 0.5% sodium deoxycholate) supplemented with freshly added protease inhibitors: 1% phenylmethylsulfonyl fluoride (PMSF, Sigma-Aldrich), 0.1% aprotinin (Sigma-Aldrich), 1 µg/mL leupeptin (Roche), and 1 µg/mL pepstatin (Biomol). Cell lysis was performed at 4°C. Protein concentrations were determined using the Bradford Reagent-based Bio-Rad protein assay (Bio-Rad). Equal amounts of total protein were resolved by SDS-polyacrylamide gel electrophoresis and transferred onto 0.45 µm nitrocellulose membranes (Amersham) via western blotting. Membranes were blocked overnight at 4°C in PBS containing 5% non-fat dry milk. Subsequently, membranes were incubated for 2 h at room temperature with the appropriate primary antibodies diluted in PBS (see Table 1). After washing, membranes were incubated for 2 h with matching horseradish peroxidase (HRP)-conjugated secondary antibodies (Table 1) in PBS containing 3% non-fat dry milk and 0.1% Tween-20 (PBS-T). Protein bands were detected by enhanced chemiluminescence using medical X-ray films (CEA RP). Autoradiograms were scanned, cropped, and processed using Adobe Photoshop CS6, and figures were assembled with Inkscape.

**TABLE 1:** Antibodies

	Antibody	Purpose (WB/IF/ChIP)^*a*^	Reference or source
Primary antibodies	α-p53 DO-1 mouse mAb	WB and IF	Santa Cruz Biotechnology
	α-p21 F-5 mouse mAb	WB	Santa Cruz Biotechnology
	α-E1A M73 mouse mAb	WB and IF	69
	α-E1B-55K 2A6 mouse mAb	WB and IF	70
	α-actin A-5441 mouse mAb	WB	Sigma-Aldrich
	α-E1B-19K rabbit pAb	WB and IF	71
	α-H3ac 06-559 rabbit mAb	WB and ChIP	Merck
	α-H3K4me3 04-745 rabbit mAb	WB and ChIP	Merck
	α-H3K9me3 39161 rabbit mAb	WB and ChIP	Active Motif
	α-H3K27me3 04-449 rabbit mAb	WB and ChIP	Merck
Secondary antibodies	α-rabbit-HRP	WB	Jackson ImmunoResearch
	α-mouse-HRP	WB	Jackson ImmunoResearch
	α-mouse Alexa Fluor 488	IF	Thermo Scientific
	α-rabbit Alexa Fluor 488	IF	Thermo Scientific
	α-mouse Alexa Fluor 555	IF	Thermo Scientific
	α-rabbit Alexa Fluor 555	IF	Thermo Scientific

^*a*^ WB, western blotting; IF, immunofluorescence.

### Immunofluorescence assays

Indirect immunofluorescence assays were performed as previously described (22). Briefly, cells were grown on glass coverslips, fixed with 4% paraformaldehyde, and blocked with TBS-BG buffer. Primary antibodies were applied for 1 h, followed by incubation with Alexa Fluor 488- or 555-conjugated secondary antibodies and DAPI (Table 1). After washing, coverslips were mounted and imaged using a Nikon A1 confocal microscope. Images were processed with ImageJ and assembled in Inkscape.

### RNA extractions

A total of 0.5 to 1 × 10⁶ cells were resuspended in 1 mL of TRIzol Reagent (Thermo Fisher), and total RNA was extracted according to the manufacturer’s protocol. To enhance RNA recovery, 3 μL of 5 mg/mL glycogen (Invitrogen) was added during the isopropanol precipitation step. Samples were washed twice with ethanol to minimize phenol contamination, then eluted in an appropriate volume of DEPC-treated water (Qiagen) and stored at −80°C. RNA integrity was assessed using the Agilent 2100 Bioanalyzer System with an RNA 6000 Nano Chip (Agilent).

### Native MNase ChIP-seq

We used the native MNase ChIP method described by Brind’Amour et al. to enrich non-cross-linked and unmasked DNA bound by histones, targeting specific protein PTMs (H3K4me3, H3K27ac, H3K27me3, and H3K9me3) (72). A total of 10^6^ cells were washed with PBS containing protease inhibitors (1 mM PMSF, 10 U/mL aprotinin, 1 μg/mL leupeptin, 1 μg/mL pepstatin, and 5 mM sodium butyrate), pelleted (500 g, 4°C, 5 min), flash frozen in liquid nitrogen, and stored at −80°C or processed immediately. Pellets were thawed on ice, resuspended in 50 μL nuclear isolation buffer with the above-mentioned protease inhibitors, and mixed by pipetting. MNase master mix (final 2 U/μL) was added, and samples were incubated at 37°C for 7.5 min. Digestion was stopped with EDTA and Triton-sodium-deoxycholate solution, then incubated on ice for 15 min and vortexed. Samples were diluted to 200 μL with immunoprecipitation buffer. Preclearing was performed with 10 μL protein A/G magnetic beads (Life Technologies) for 6 h at 4°C. Simultaneously, antibody-bead complexes were prepared by incubating 10 μL beads with 1 μg antibody in 100 μL immunoprecipitation buffer for 6 h at 4°C. Precleared chromatin was added to antibody-bead complexes of 10^5^ cells per reaction and incubated overnight at 4°C. Beads were washed twice with low-salt buffer, once with high-salt buffer, and DNA was eluted with 30 μL elution buffer at 65°C for 1.5 h. Eluates were combined, phenol-chloroform extracted, and DNA precipitated overnight with NaCl, glycogen, and ethanol at −80°C. After centrifugation, pellets were washed with 70% ethanol, dried, and resuspended in 30 μL EB buffer at 37°C for 1–2 h. DNA was stored at −20°C until sequencing. Chromatin digestion efficiency was checked by agarose gel electrophoresis of input samples.

### Library preparation and HTS

Library preparation and HTS were performed exactly as previously described (22). Briefly, ChIP-DNA libraries were prepared by the in-house sequencing facility using the NEXTflex ChIP-seq Kit (PerkinElmer Applied Genomics), following the manufacturer’s instructions. RNA libraries were similarly prepared by the same facility using the NEXTflex Rapid Directional RNA-seq Kit (PerkinElmer Applied Genomics), according to the manufacturer’s protocol.

### Sequencing data analyses

In short, for ChIP-seq, histone PTM peaks from individual biological replicates were called via MACS2 (H3K4me3 and H3K27ac) or epic2/SICER (H3K27me3 and H3K9me3) versus their respective input (73, 74). MSPC was used on the replicates to identify the true peaks and discard false-positive calls (75). Statistically significant differences between the E1B-55K (+) and (−) conditions were calculated via the diffReps software (76). These differential regions were then overlaid with the MACS2/MSPC- or epic2/MSPC validated peaks to generate a final set of peaks that were used in this work. All annotation of the peak data was done via the ChIPseeker R package (77). For RNA-seq, we used the DESeq2 R package (78) to quantify any statistically significant differences on the RNA-seq expression changes between the E1B-55K (+) and (−) conditions. The EaSeq software (easeq.net) has been used to create all genome tracks in this work (79). The sequencing data analysis is visualized for clarity in Fig. S1. All associated source data can be found in the source data Excel sheet. The DNA alignment, peak calling, and validation, as well as the RNA-seq transcript quantification script, were uploaded to the Zenodo open access repository and can be found at doi.org/10.5281/zenodo.17553960. Likewise, the downstream data analysis scripts can be found at doi.org/10.5281/zenodo.17457625 for ChIP-seq and doi.org/10.5281/zenodo.17457811 for RNA-seq, respectively.

### Statistical analyses

The Pearson correlation coefficient calculations shown in Fig. 3B were performed by correlating the magnitude of RNA-seq expression changes (log₂ fold change) with the corresponding promoter-associated ChIP-seq signal changes (log₂ fold change) for significantly altered genes (adjusted *P* < 0.01 and log₂ fold change < −0.5 or >0.5).

## Data Availability

Raw sequencing data were deposited in the European Nucleotide Archive (ENA) under accession PRJEB102597 (https://www.ebi.ac.uk/ena/browser/view/PRJEB102597).

## SUPPLEMENTAL MATERIAL

Supplemental material mbio.03470-25-s0001.pdf Figures S1 and S2.

Source data mbio.03470-25-s0002.xlsx Raw and processed ChIP-seq and RNA-seq data used in this study.

ASM does not own the copyrights to Supplemental Material that may be linked to, or accessed through, an article. The authors have granted ASM a non-exclusive, world-wide license to publish the Supplemental Material files. Please contact the corresponding author directly for reuse.
