# Supplementary material for: The adenovirus oncoprotein E1B-55K reshapes epigenetic histone modifications in primary human cells
Source: mBio. 2026 Jan 26;17(3):e03470-25. doi: 10.1128/mbio.03470-25 (PMC12977564; doi:10.1128/mbio.03470-25)
Supplement: Supplemental material — Figures S1 and S2. [file mbio.03470-25-s0001.pdf]

Supplementary information

The adenovirus oncoprotein E1B-55K reshapes epigenetic histone modifications in primary human cells

von Stromberg *et al*.

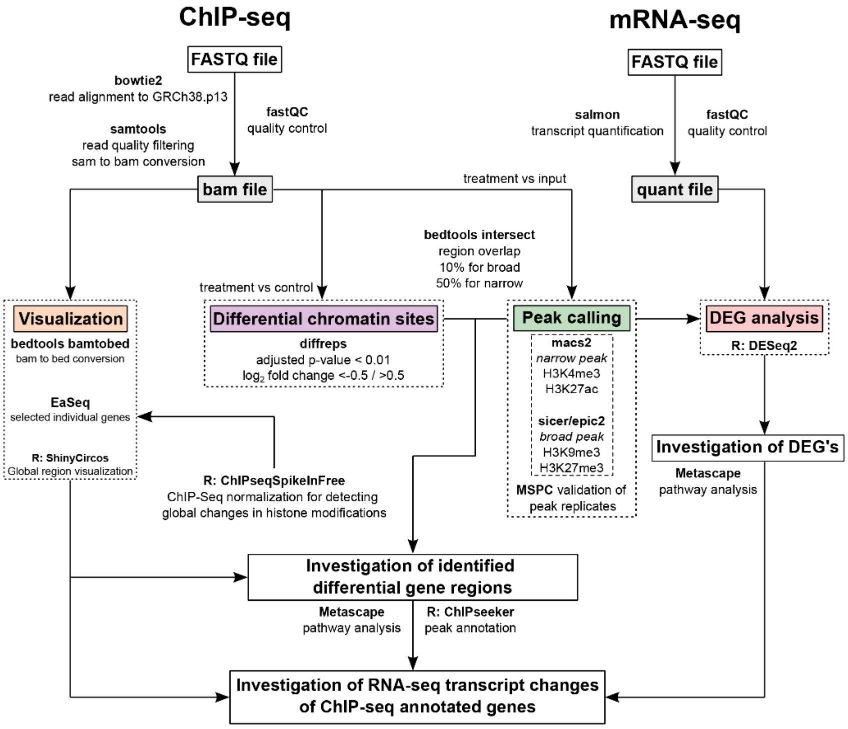

Figure S1: Bioinformatics workflow.

Flowchart illustrating the bioinformatics software and tools used for DNA sequence alignment, narrow peak calling, data visualization, and functional analysis with R packages, as well as software employed for differential gene expression analysis of mRNA sets.

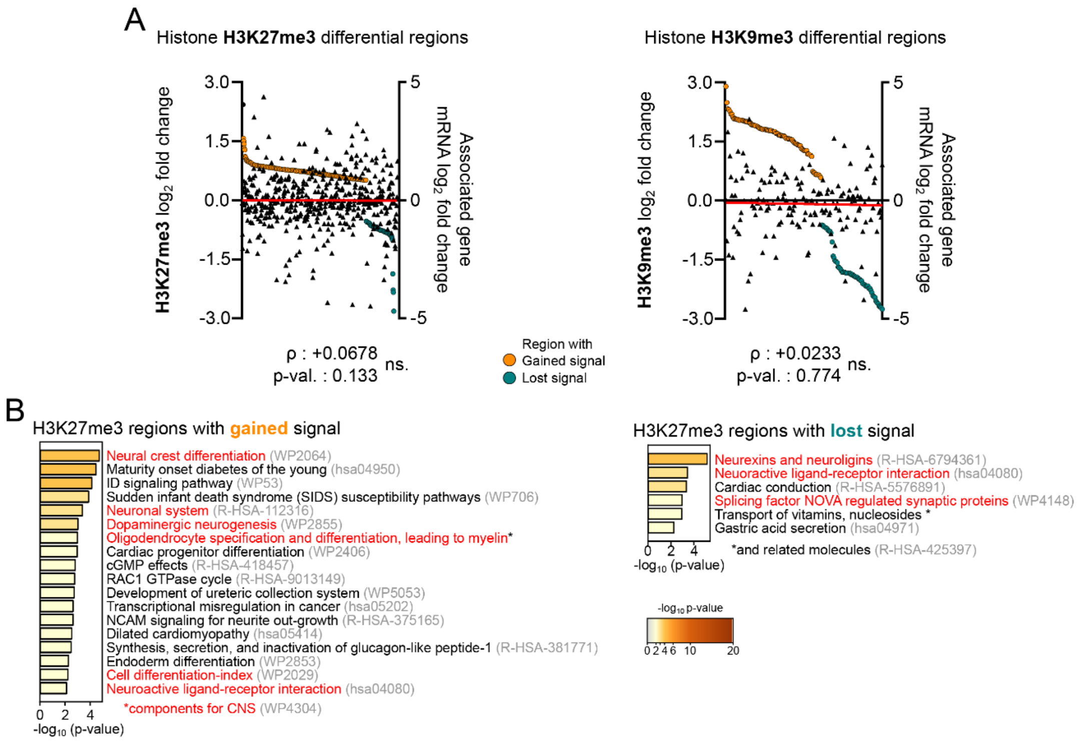

Figure S2: Influence of E1B-55K on heterochromatin-associated histone PTM.

(**A**) Quantitative correlational analysis between altered histone PTMs and associated gene expression changes (up- or downregulation). The Pearson correlation coefficient (ρ) represents the strength and direction of the linear relationship between these two continuous variables. P-values were calculated using a two-tailed t-test. ns: not significant ($P > 0.05$).

(**B**) Metascape pathway enrichment analysis of genes associated with H3K27me3 histone marks gain (left) or loss (right). Red-highlighted pathways are related to neuronal cell differentiation.
